# Correction: Subspheroids in the lithic assemblage of Barranco León (Spain): Recognizing the late Oldowan in Europe

**DOI:** 10.1371/journal.pone.0231036

**Published:** 2020-03-23

**Authors:** The PLOS ONE Staff

There are errors in the Author Contributions. The publisher apologizes for the errors. The correct contributions are:

**Conceptualization:** Stefania Titton, Deborah Barsky
**Data curation:** Stefania Titton, Deborah Barsky, Amèlia Bargalló
**Formal analysis:** Stefania Titton, Alexia Serrano-Ramos
**Funding acquisition:** Juan Manuel Jimenez Arenas
**Investigation:** Stefania Titton, Deborah Barsky, Josep Maria Vergès, José García Solano
**Methodology:** Stefania Titton
**Project administration:** Isidro Toro-Moyano, Robert Sala-Ramos, Juan Manuel Jimenez Arenas
**Resources:** Isidro Toro-Moyano
**Software:** Alexia Serrano-Ramos
**Supervision:** Deborah Barsky, Amèlia Bargalló
**Visualization:** Stefania Titton
**Writing—original draft:** Stefania Titton
**Writing—review & editing:** Deborah Barsky, Amèlia Bargalló, Juan Manuel Jimenez Arenas
