# Supplementary material for: Subspheroids in the lithic assemblage of Barranco León (Spain): Recognizing the late Oldowan in Europe
Source: PLoS One. 2020 Jan 30;15(1):e0228290. doi: 10.1371/journal.pone.0228290 (PMC6992009; doi:10.1371/journal.pone.0228290)
Supplement: Attachment — Submitted filename: Paper Review.pdf [file pone.0228290.s004.pdf]

The paper presents a new addition on the lithic assemblage from a well-known European Mode 1 site, Barranco Leon. Specifically, it focuses on a small subspheroid assemblage made on limestone that has been analyzed through a four-phase technological perspective. I was excited for reviewing this paper, but after finished I'm left with the feeling that more could have been done. Although the general data presentation is correct, I find a huge scarcity on some of the paper sections, especially on the one related to the Morphometric analysis, which to me should have been the biggest asset of the paper. Therefore I recommend this paper to go through major revisions

I strongly recommend a general English revision of the manuscript. Maybe the authors can ask an English native colleague to review it for them. In addition, here is a list of some of the writing mistakes I was able to find during my revision:

Lin.156: "we was apply"

Lin. 268: "to remedy to the difficulties"

Lin. 176: "edorrheic"

Lin. 773: "be may"

Lin. 447: "visibles"

Lin. 613: "anges"

Lin.799: "Sometimes the use of stone anvil attested"

Lin. 835: "recongnized"

INTRODUCTION

Lin. 67: "*… has been tentatively attributed to* Kenyapithecus*…"*

I've never seen the word *Kenyapithecus* published anywhere. *Kenyanthropus platyopts* is the name given to the species in its original paper and it's also how appear in the several publications that mention it. To mention a couple:

Leakey, M. G., Spoor, F., Brown, F. H., Gathogo, P. N., Kiarie, C., Leakey, L. N., & McDougall, I. (2001). New hominin genus from eastern Africa shows diverse middle Pliocene lineages. *Nature*, *410*(6827), 433.

Spoor, F., Leakey, M. G., & O'Higgins, P. (2016). Middle Pliocene hominin diversity: Australopithecus deyiremeda and Kenyanthropus platyops. *Philosophical Transactions of the Royal Society B: Biological Sciences*, *371*(1698), 20150231.

Lin 78-79: *"… generating a process of distinction from other primates, underlined by the development of technical skills."*

It's probably due to the way this sentence is written, but it looks that the authors are suggesting the mentioned process of distinction is somehow a conscious one. Furthermore, enough evidences of technology in chimpanzee contexts have been provided, so I would try to be careful with this kind of statements.

Lin. 87-91: *"… Through time, the transition towards new reduction systems occurred within the OIC [28], progressively moving beyond the mechanics of the unifacial and unidirectional strategies, and leading to greater morpho-technological variability within some of the Oldowan tool kits, with orthogonal, unifacial discoid and multidirectional core management strategies…"*

This statement is something to take into consideration. Is the suggested transition a real transition? Advanced Oldowan reduction strategies can be found in OGS-7, Lokalalei 2C and Kanjera South, to mention some, and all those sites predate 2.0 Ma. The Oldowan manifests a large regional variability in its complexity, both synchronic and diachronic, so I find risky, to say the least, the suggestion of a lineal progressive evolution. It doesn't seem to be supported by the current state of the art. I know the authors have quoted this paper later on the text, but I find it essential in order to work through this paragraph.

Stout, D., Semaw, S., Rogers, M. J., & Cauche, D. (2010). Technological variation in the earliest Oldowan from Gona, Afar, Ethiopia. *Journal of Human Evolution*, *58*(6), 474-491.

Lin. 118 – 121: *"Based on these descriptions, the different categories comprising the PSSB can be distinguished from each other by direct observation in order to recognize deviances from the regular morphological-volumetric aspect of a sphere, developed around a central point (center of mass), which confers them a more or less rounded shape."*

To me, that is just a way to recognize the inherent subjectivity of any non-quantitative lithic analysis.

MATERIALS AND METHODS

Lin. 258: "*Cores are scarce and intensively reduced (3.1 % of the 1 562 pieces in flint)"*

How did the authors measure the reduction intensity of the cores? Which are the knapping strategies used? Do they correlate with the ones identified on the limestone assemblage? I feel like there is very few explanation regarding this topic, while a lot of information (maybe not that necessary) is provided on the general characteristics of the assemblage. I also get that is not exactly the point of this paper to present the core features, but they are the elements that could possibly be correlated with the following PSSB discussion.

Lin. 265: *"After reviewing the entire lithic collection, five limestone pieces…"*

The PSSB pieces sample is really small, five in total, a 0.2% of the whole lithic collection. I'm fully aware that there is nothing the authors can do about it; the sample is what it is, but they should be aware of the problem this sample poses if they intend to include the BL subspheroid assemblage into the large, pancontinental Oldowan discussion.

Lin. 268-271: *"To remedy to the difficulties relating to surface preservation of these five pieces and to recognize and verify their special features and assess the direction of their removal negatives, we decided to compare them with 2 of the best preserved multifacial multipolar cores (out of the 10 available in the collection)"*

I don't fully understand why the authors did this. Establishing a comparison with multifacial multipolar cores implies that they thought about a relation between the two types of artifacts before the actual analysis of the PSSBs. If that is the case, I don't understand why they limited the comparison to only two cores of the total assemblage, if taking all of them would have helped to enlarge the sample and obtain more reliable data. These also relates with my previous comment on why the authors didn't provide with a larger explanation on the core's features.

RESULTS

Lin.447-450: *"For the pieces with visibles impact points (N=number of angle with impact point visible for each piece), we calculated the average of the knapping angles (kn.a): Tools D and E (134 °, N kn.a = 7 and 131 °, N kn.a = 8, respectively) showed more acute angles than for Tools F and G (121 °, N kn.a = 9 and 116 °, N kn.a = 4, respectively)."*

How can 134 and 131 degrees angles been more acute than 121 and 116 degrees angles?

Lin. 451-453: *"While the final morphologies of the tools occur as cuboid, polyhedral, polyhedral-rounded or rounded, the analysis of residual cortex indicates a priori anthropic selection of rounded cobbles"*

These statement necessarily implies that all the selected pieces are volumetrically reduced at the exact same stage, which they don't know or at least haven't explain anywhere on the paper.

Lin. 459-460: "The diachritical analysis allowed us to reconstruct each phase of manufacture for four of the tools (Tools D, E, F, G) (Fig 7, Fig 8, Fig 9, and Fig 10)"

It might only be a matter of semantics, but I find that saying "each phase" might be a little bit risky. As they didn't evaluate the reduction stage of the pieces, and assuming that new flake removals tend to erase the previous ones, I think it would be more accurate to say that they recognized the last phases of the sequence, not each one of them.

Lin. 467: "Tool D (Fig 7)…"

I don't really see how the yellow surface presented in Profile E correlate with the green one presented in profile B, so its sequential ordination seems unclear to me.

Also, if a percussion platform is been created in Phase 1 (green), how the percussion platform for Phase 2 in Profile E appears to be the blue one (associated a Phase 3)? One possible explanation could be that the previous presence of Phase 1 removals were erased by the posterior Phase 3, but I cannot assure it just with pictures nor with the quality of the 3D scans. In any case, Figure 7 does not result very clear, and I recommend the authors to provide a more detailed explanation of the relationship between surfaces and Phases.

Lin. 492: "Tool E (Fig 8)…"

In Profile A, why aren't the Phase 1 removals numbered? I don't see any identification problem and I believe are the same ones marked on Profile F.

Lin. 511: "Tool F (Fig 9)…"

In general, not only on this tool, I think the graphic design on the diachritical schemes should be improved, as sometimes is leading to misinterpretations. For example, in Profile E, Phase 2b (dark yellow), the scar marked as 6* is clearly cutting into the one marked as 8*. Therefore, one would say that scar is posterior to 8* and not a previous one, when, judging by the actual photo and the authors explanation that is not the case.

Lin. 643-645: *"This characteristic of the second group gives the objects a more rounded shape than those of the first group, which, contrastingly, tends to present more angular and irregular profiles".*

That is a little bit obvious, isn't it? I really appreciate the intention of been thorough and pursue a quantitative approach with this paper, but the statement that a spherical volume has less acute angles than a polyhedron is hardly a result. In general terms, the morphometric analysis here presented looks a little bit scarce. These section of the paper is crucial to me and should be extended.

DISCUSSION

Lin. 709: *"When the PSSB are accompanied by Acheulian elements in an assemblage, their cultural attribution is moved from Oldowan to Acheulian (Table 4)".*

Well, of course. It is a set of artifacts displaying diachronic presence. What is the point of remarking the artificial nature of any taxonomic category? Especially when those categories doesn't take in account actual technological features, complexity parameters or quantitative data, but presence/absence of recognizable tools.

Lin. 718-719: *"Contrastingly, in sites attributed to the Oldowan, PSSB are associated with percussive implements and there are no handaxes".*

This is, again, an example of circular reasoning that doesn't say anything about the actual PSSBs.

Lin. 745-747: *"The ability of hominins to pursue a recurrent strategy in the management of multifacial multipolar cores, as in the Orce subspherods described here, represents a step forward compared to the older assemblages in terms of cognitive advancement"*.

There are older chronologies with long, systematized knapping sequences (See again Lokalalei 2C or even Gona). I don't really see the step forward if we are referring to a set of lithic artifacts largely heterogenic that doesn't present clear attributes of a final morphology. I find difficult to refer "PSSB" as a morphotype. It is a term that widely refer to diversity, but the elements that comprise them are quite different. So far, I've not seen any attempt to explain why that variability appear on the archaeological record.

Lin. 781-784: *"We define polyhedron morphologies by higher variability in their angles (Fig 13), which are also more acute, likening them to the group of multifacial cores. Comparatively, truly subspheroid morphologies display more open facet angles and greater uniformity"*.

Again, this statement relates with my previous comment on subspheroids being more rounded than polyhedrons on the Results section.
