# Supplementary material for: Subspheroids in the lithic assemblage of Barranco León (Spain): Recognizing the late Oldowan in Europe
Source: PLoS One. 2020 Jan 30;15(1):e0228290. doi: 10.1371/journal.pone.0228290 (PMC6992009; doi:10.1371/journal.pone.0228290)
Supplement: Attachment — Submitted filename: Review.docx [file pone.0228290.s003.docx]

**Review: Subspheroids in the lithic assemblage of Barranco León (Spain): recognizing the late Oldowan in Europe**

This study presents some important findings on spheroids artifacts: it shows that these subspheroids were the product of a systematic management of rounded cobbles (line 829); polyhedron morphologies van be defined by higher variability in their angles, which are also more acute (783) and these morphologies in the BL lithic assemblage do not result from continuous battering (786).

The conclusion states: In (the) future (846), it will be interesting to analyze larger samples of PSSB morphologies using the methodology presented here, in order to shed light on (the) hypothesis about whether such forms derive from the interest of obtaining a certain volume around a central point. Dispersion of the mass with respect to this point could indicate differences between the categories.

However, why this hypothesis should be of importance is not fully discussed. The most effective solid projectile has a spherical form in which the most mass can be concentrated into the least dimension, so this hypothesis is assumed to include the probable use of these tools as projectiles.

The mass of the tools is given in Table 2 is consistent with examples from the natural spheroids of the Cave of Hearths and which have been shown to be good throwing material in terms of impact energy and muscle power (Wilson et al 2016, Cannell 2002, Cannell 2018), with an average of 502 g – similar to the Cave of Hearths value of 533 g. Although the number of samples does not allow for a mass distribution analysis, it would be of interest to examine their mass, together with that of the 51 percussion tools (active and passive) and 249 pebbles and cobbles without anthropic traces. Indeed, given the close proximity of the Fuente Nueva site and similarity of the limestone cobbles (whole and broken) and cores, (which make up about 75% of the 446 macro objects- tools and possible manuports) of the combined assemblage (ref. 48), the mass distribution of the subspheroids in relation to the two assemblages could add greatly to our comprehension of these tools.

This is of particular importance given the evidence for confrontation between hominins and large carnivores at Fuente Nueva, where an incomplete skeleton of *Mammuthus meridionalis* was found surrounded by 34 coprolites, 17 lithic artifacts, and 32 unmodified cobbles. The skewed spatial distribution of these elements, the physical characteristics of the coprolites, and the absence of the elephant limbs and cranium suggest that both hominins and hyenas scavenged the carcass of this megaherbivore, following a sequence of consumption in which the hominins arrived first, dismembered and transported the limbs, and probably also the cranium, and later the hyenas consumed the rest of the elephant carcass (Espigares et al., 2013).

It would also be of interest to note that similar Olduwan spheroids have been found at Ain Hanech, Algeria (Early Pleistocene and dated at about 1.8 Ma) and Hummal, Syria (in deposits dated to at least 1 Ma). In the former case the classification of many rocks as spheroids has been disputed; originally described as “sphéroides à facettes” or faceted spheroids (Balout, 1955), a subsequent analysis suggested that “the bulk of the assemblage was comprised of simple cores,” (Sahnouni, Schick, & Toth, 1997, p. 703). The present work and results of the close examination of the angles used may help to clarify the cataloguing of this largest assemblage of spheroid material.

At Hummal, two distinct mass distributions were found, one: «les gros, dont le diamètre est d’environ 8 cm pour un poids moyen de 540 g; les petits, d’un diamètre autour de 5,5 cm pour un poids moyen de 150 g.” The spheroids of Barranco are all in the former group, but there may be some samples that also fit this lighter mass, adding to the evidence of a single species selection with specific age or sex distributions.

To give more context to the site, it would help to give the full coordinates and presumed height above the lake surface. Level D1 is said to materialize a high-energy, rapid water transport of gravels and cobbles (198) so presumably this flood level was still well above the lake surface and the site protected at the rear by the rock face (?), but with open approaches that would need defense. This is of importance as it would allow another level of comparison with other sites where spheroid-like artefacts have been found.

Reference 109 is given as the source for examples at Ain Boucherit, Algeria, but it is surely worth mentioning the dating of the site of large polyhedrons at about 2.4 Ma, only 750 km distant from Barranco Léon, but separated by an immense time difference.

Some specific points (highlighted in red):

The naming of these tools should be constant and in the same case:

Fig 2. Polyhedral and subspheroid morphotypes from the BL site (a-e), and 280 multidirectional cores selected for the diachritical analysis (f-g).

455 Tools listed A to F all present a polyhedron or subspheroidal morphology, Tools G and H are polyhedral cores

From Fig. 2 and the diacritical analysis it appears that the cores are actually tools a and b (A and B) (?)

545 cortical surfaces, attributed to active percussion (Profile C and E). The use of this tool for (546) percussion in (is) posterior to the previous phases of management

561 temporal order within the management phase: 1) a knapping phase and 2) a pounding (562) phase (indicated in yellow and purple, respectively, Fig 11.). The figure uses the word ‘percussion’ rather than pounding, which implies hand-held striking. The marks on the rock’s angles could certainly be the result of several thrown impacts (training) and are unlike the (what appears to be) pounding marks in Fig. 10. The text states: (260) the limestone was used for different purposes; all mainly linked to percussive activities (261).	A thrown rock is also percussive in that impact marks will show if it has been thrown and hit or landed on a hard surface.

721 Bisi and colleagues [100] found on the surface at Ca 'Belvedere de Monte Poggiolo (Ca’ Belvedere)

799 Sometimes the use of stone anvil attested (Fig 7)…. (Text missing ??)

802 innovation [188]. This justifies some DOB sites being reaassigned Acheulian de la Torre 803 [137]. (to the as stated by?)

**Suggested Additional References**

Wilson, A. D., Zhu, Q., Barham, L., Stanistreet, I., & Bingham, G. P. (2016). A dynamical analysis of the suitability of prehistoric spheroids from the cave of hearths as thrown projectiles. Nature, Scientific Reports, 6, 323.

Cannell, A. (2002). Throwing behaviour and the mass distribution of geological hand samples, hand grenades and olduvian manuports. Journal of Archaeological Science, 29, 335–339.

Cannell Alan (2018): Mass Distribution Analysis of Spheroid Manuports, Spheroid Artifacts, and the Lithics of Play Learning, Lithic Technology, DOI: 10.1080/01977261.2018.1460703

Espigares, M. P., Martínez-Navarro, B., Palmqvist, P., Ros-Montoya, S., Toro, I., Agustí, J., & Sala, R. (2013). Homo vs. Pachycrocuta: Earliest evidence of competition for an elephant carcass between scavengers at Fuente Nueva-3 (Orce, Spain). Quaternary International, 295, 113–125.
