# Supplementary material for: Subspheroids in the lithic assemblage of Barranco León (Spain): Recognizing the late Oldowan in Europe
Source: PLoS One. 2020 Jan 30;15(1):e0228290. doi: 10.1371/journal.pone.0228290 (PMC6992009; doi:10.1371/journal.pone.0228290)
Supplement: Attachment — Submitted filename: SECOND REVIEW.docx [file pone.0228290.s006.docx]

SECOND REVIEW:

The authors have successfully resolved most of the issues highlighted in the previous revision. I really appreciate the effort made on the manuscript edition, and the Introduction, Context of BL, Materials and Methods sections are relatively clear to my view. I also consider very positive the inclusion of the Supporting Information File in the morphometric analysis.

However, the Results section remains a little problematic to me. As I mentioned in the previous revision, the diacritical schemes presented are not as clear as they should be, and could easily be misinterpreted, probably due to the type of drawing. The authors have made minor changes in Figures 7 and 8, but I still feel that more effort could be made regarding the informative content and aesthetics of the Figures. There is no disagreement with the authors on the way the phases have been identified; just the way this information has been presented.

I would also like to discuss a bit more the relationship between the subspheroid sample analysed and the multifacial cores. Furthermore, I suggest the authors to provide a deeper explanation about *why* they chose to compare the PSSBs in the first place. During the whole manuscript, the two multifacial multipolar cores have been considered as part of the PSSBs assemblage, or at least that is the impression I got, despite this paragraph included in the Materials and Methods section in which the authors say that are different things:

“*After reviewing the entire lithic collection, five limestone pieces (0.2 % of the whole 294 collection) were classified by their morphological characteristics and special technological features as attributable to the PSSB group as defined by Kleindienst [35], Leakey [13] and 296 more recently by Tixier & Roche [34]. To overcome the difficulties relating to surface preservation of these pieces and to recognize and verify their special features and assess the direction of their removal negatives, we decided to compare them with 2/10 of the multifacial multipolar cores. In fact, only these two cores presented surface conservation sufficient to realize a diacritical study (visible impact points and removal directions)”.*

I think this is a key point on the paper, specially taking in account that the diacritical schemes have been fully performed in 2 of the 5 subspheroids (D and E) and in the 2 multifacial multipolar cores (F and G). The recognition of the same management phases in artefacts that have been classified as different is worth detailed explanation and further comment.

In the Discussion section, the authors comment: “*the diachritic analysis has allowed us to evaluate that, at least for the BL site, sub-spheroid morphologies result from a well-reasoned, organized shaping process that sometimes involved the use of a stone anvil (Fig 7). Meanwhile, such preconceived morphological templates transposed onto stone are usually attributed to the Acheulian, concretized, for Isaac [191], by the presence of innovative handaxes and standardized tools with recurring morphologies”.* Again, I suggest being careful with this kind of statements, and this is the reason: the diacritical analysis performed in Tools D and E show what appears to be a shaping phase, supporting the author’s statement. I cannot really value Tool C, as its diacritical scheme is partial. Also, I’m not including the results coming from Tools F and G as the previous paragraph strictly refers to “*sub-spheroid morphologies”* and, as I mentioned, the relation of the multifacial multipolar cores with the PPSBs remains a bit confusing to me. The other two PPSBs (Tools A and B) were discarded from the analysis due to a “*high degree of alteration”* (Lin. 504).

In the end, from the initial 5 PPSBs and 2 cores included in the original sample, only two of them (Tools D and E) show evidence of shaping. Again, I am not disagreeing with the authors; I just want to clarify why I think that the general interpretation of these results has to be cautious. I think the original sample is limited and extrapolations made from two artefacts can be problematic.

I do not consider the things I have pointed out as Major Revisions. There are very specific issues that are easily solvable. However, I consider necessary feedback in each one of them before seeing this paper successfully published.

Again, I want to thank the authors for the time and effort they put in correcting the previous version of this manuscript.
