# Supplementary material for: Subspheroids in the lithic assemblage of Barranco León (Spain): Recognizing the late Oldowan in Europe
Source: PLoS One. 2020 Jan 30;15(1):e0228290. doi: 10.1371/journal.pone.0228290 (PMC6992009; doi:10.1371/journal.pone.0228290)
Supplement: Attachment — Submitted filename: Response to Reviewers.docx [file pone.0228290.s007.docx]

SECOND REVIEW:

The authors have successfully resolved most of the issues highlighted in the previous revision. I really appreciate the effort made on the manuscript edition, and the Introduction, Context of BL, Materials and Methods sections are relatively clear to my view. I also consider very positive the inclusion of the Supporting Information File in the morphometric analysis.

However, the Results section remains a little problematic to me. As I mentioned in the previous revision, the diacritical schemes presented are not as clear as they should be, and could easily be misinterpreted, probably due to the type of drawing. The authors have made minor changes in Figures 7 and 8, but I still feel that more effort could be made regarding the informative content and aesthetics of the Figures. There is no disagreement with the authors on the way the phases have been identified; just the way this information has been presented.

We agree with the Reviewer here and we are very grateful for this remark as it has allowed us to make a far better visual presentation of our schemes. The pieces have been redesigned improving their graphic representation.

I would also like to discuss a bit more the relationship between the subspheroid sample analysed and the multifacial cores. Furthermore, I suggest the authors to provide a deeper explanation about *why* they chose to compare the PSSBs in the first place.

To better explain and emphasize the concept we added in the manuscript the sentence underlined in yellow.

During the whole manuscript, the two multifacial multipolar cores have been considered as part of the PSSBs assemblage, or at least that is the impression I got, despite this paragraph included in the Materials and Methods section in which the authors say that are different things:

In answer to ‘why’ we chose to compare PSSBs in the first place:

1) We had an assemblage containing pieces that were evidently reduced using the same strategy: multifacial multipolar.
2) Some of these pieces presented a spheroid morphology (i.e. they were more rounded).
3) We needed, therefore to elaborate a scientific and objective methodology in order to check our determination that these pieces were really different from the multipolar multifacial cores.

To further clarify this in the text selected by the reviewer, we added the highlighted phrases indicated as follows:

“*After reviewing the entire lithic collection, five limestone pieces (0.2 % of the whole 294 collection) were classified by their morphological characteristics and special technological features as attributable to the PSSB group as defined by Kleindienst [35], Leakey [13] and 296 more recently by Tixier & Roche [34]. W*e compare the pieces that share the multifacial and multidirectional management strategies, including those with semi-rounded/rounded morphologies (the PSSB), using diacritical analysis. In order to evaluate whether or not the PSSB can be isolated with respect to the cores, a morphometric analysis and statistical was carried out. *To overcome the difficulties relating to surface preservation of these pieces and to recognize and verify their special features and assess the direction of their removal negatives, we decided to compare them with 2/10 of the multifacial multipolar cores. In fact, only these two cores presented surface conservation sufficient to realize a diacritical study (visible impact points and removal directions)”.*

Cores are not defined as PSSB, but are included in the text and in figure 2 as a comparative sample. But, in order to eliminate the subjectivity in the subdivision of categories, during the analysis, the sample was evaluated as a whole. In fact, the 7 pieces are described in the results section as: “...*multipolar and multifaceted limestone tools*...” all of them presenting this characteristic (but distinguishable by their different morphologies). In the analysis: surface alteration; dimensional features (here we further underlined in the Table 2: *Tools F and G are polyhedral cores used for comparative purposes*); diacritical study, percussion marks analysis, and in the morphometric analysis as well, the pieces are all named as **tools** and not defined or included in a PSSB type assemblage.

The statistical/morphometric analysis allows us to re-divide the sample into two groups (A and B): *“Although the Lubishew’s test indicates only a moderate degree of discrimination between groups, descriptive statistics highlights a morphological distinction between the forms, linked to the width of the angles.”*

Group A is different from B (spheroid and subspheroid morphologies) and is characterized by tools with more acute angles with higher variability in their amplitude (Table 3 and Figure 13), in which we can find multifaceted and multipolar cores (with irregular morphology) and the polyhedral PSSB subcategory.

*“In this study, the differentiation between polyhedrons, subspheroids and spheroids is not, therefore, based on criteria relating to raw materials, but rather has been made using the width of the angles separating the facets of each piece. We define polyhedron morphologies by higher variability in their angles (Fig 13), which are also more acute, likening them to the group of multifacial cores. Comparatively, truly subspheroid morphologies display more open facet angles and greater uniformity.”*

We therefore take into account throughout the manuscript, even if all the pieces are included in the same analysis, the distinction between the PSSB and multipolar cores. As well as the subcategories PSSB: polyhedron, spheroids and subspheroids.

We hope therefore, that the sentences added in the text (indicated in yellow) will make the concept clearer.

I think this is a key point on the paper, specially taking in account that the diacritical schemes have been fully performed in 2 of the 5 subspheroids (D and E) and in the 2 multifacial multipolar cores (F and G). The recognition of the same management phases in artefacts that have been classified as different is worth detailed explanation and further comment.

In the Discussion section, the authors comment: “*the diachritic analysis has allowed us to evaluate that, at least for the BL site, sub-spheroid morphologies result from a well-reasoned, organized shaping process that sometimes involved the use of a stone anvil (Fig 7). Meanwhile, such preconceived morphological templates transposed onto stone are usually attributed to the Acheulian, concretized, for Isaac [191], by the presence of innovative handaxes and standardized tools with recurring morphologies”.* Again, I suggest being careful with this kind of statements, and this is the reason: the diacritical analysis performed in Tools D and E show what appears to be a shaping phase, supporting the author’s statement. I cannot really value Tool C, as its diacritical scheme is partial. Also, I’m not including the results coming from Tools F and G as the previous paragraph strictly refers to “*sub-spheroid morphologies”* and, as I mentioned, the relation of the multifacial multipolar cores with the PPSBs remains a bit confusing to me. The other two PPSBs (Tools A and B) were discarded from the analysis due to a “*high degree of alteration”* (Lin. 504).

Beyond the discussion we have already described similarities as differences in management phases in artifacts that have been classified as different: section Results (lines 619-652) and with figure 12. But, to fill this doubt, we decided to integrate in the discussion the following sentence:

The concept of acquisition and transmission reflected in any systematic production of forms is qualified as culture. Even though the creation of a platform and subsequent peripheral management of a surface may seem simplistic, as in the case of orthogonal knapping, it became emblematic of Oldowan culture [135]. The subspheroid morphologies and the multidirectional cores of BL, for their repetition in the first phases of the management, can’t be fit in the classic opportunistic flake production. Furthermore, we have to take in account that, for the site under consideration, not only the two subspheroids (Tools D and E) show evidence of shaping, because other intentionally repeated morphotypes are present: as for the case of heavy-duty scrapers which have been defined also by intentional shaping [135]. This feature becomes more characteristic from the Acheulian techno-complex.

In the end, from the initial 5 PPSBs and 2 cores included in the original sample, only two of them (Tools D and E) show evidence of shaping. Again, I am not disagreeing with the authors; I just want to clarify why I think that the general interpretation of these results has to be cautious. I think the original sample is limited and extrapolations made from two artefacts can be problematic.

In answer to this query: we believe that although we have a limited sample, the presence of morphologies with repeated manufacture allows us to state, while using all the necessary caution, that there is a mental preconception in this hominin cultural group. Looking to other publications on the Oldowan, such preconceived morphotypes are always present in limited numbers: retouched denticulate tools, heavy-duty scrapers or ‘loosely configured tools’ (51). For example, in BL there are only 7 heavy-duty scrapers and these tools are also very scarce in other Oldowan sites where they have been identified. We argue that this is really one of the main characteristics of the Oldowan that changes later in the Acheulian, when recognizable standardization: real ‘tools’ are more numerically represented and therefore more easily discernible. Although there are only 2 pieces with optimal preservation that allow us to fit them perfectly into the subspheroid morphotype, we cannot ignore their presence.

I do not consider the things I have pointed out as Major Revisions. There are very specific issues that are easily solvable. However, I consider necessary feedback in each one of them before seeing this paper successfully published.

We hope to have been clear in our answers, and to have resolved any remaining doubts on behalf of the reviewer.

Again, I want to thank the authors for the time and effort they put in correcting the previous version of this manuscript.

We thank the Reviewer for his active contribution to the improvement of our work.
